# Development and Preclinical Evaluation of a Dual-Drug Implant for Long-Acting HIV Prevention and Contraception

**DOI:** 10.3390/pharmaceutics18091060

**Published:** 2026-08-26

**Authors:** Archana Krovi, Leanna Levin, Greg J. Gatto, Ellen H. Luecke, Rhonda Brand, Amanda Swistok, Mackenzie L. Cottrell, Amanda P. Schauer, Leah M. Johnson

**Affiliations:** 1 RTI International, 3040 E. Cornwallis Road, Research Triangle Park, Durham, NC 27709, USA; akrovi@rti.org (A.K.);; 2 Department of Medicine and Magee-Womens Research Institute, University of Pittsburgh, Pittsburgh, PA 15213, USA; 3 Division of Pharmacotherapy and Experimental Therapeutics, Eshelman School of Pharmacy, University of North Carolina, Chapel Hill, NC 27599, USA

**Keywords:** long-acting, multipurpose prevention technology, HIV PrEP, contraception, implants, combination therapy, subcutaneous

## Abstract

**Background/Objectives**: Multipurpose prevention technologies (MPTs) that combine protection against HIV and unintended pregnancy can improve women’s health outcomes by simplifying use and enhancing adherence. We developed a long-acting (LA) implant for the simultaneous and sustained delivery of the antiretroviral islatravir (ISL) and the contraceptive hormone etonogestrel (ENG). **Methods**: Using extruded poly-ε-caprolactone (PCL) tubing, reservoir-style implants were fabricated and evaluated in three configurations: (Group 1) a two-segment implant formed by joining separate ISL- and ENG-containing tubes; (Group 2) a single tube divided into two drug compartments by a heat-sealed PCL spacer; (Group 3) separate single-drug implants. In vitro release was assessed in phosphate-buffered saline at 37 °C, and safety and pharmacokinetic (PK) profiles were evaluated in New Zealand White rabbits (n = 4/group) over approximately 90 days. **Results**: The average ISL daily in vitro release rates for Groups 1, 2, and 3 were 29 ± 5 µg/day, 18 ± 5 µg/day, and 47 ± 8 µg/day, respectively, and ENG daily in vitro release rates were 39 ± 9 µg/day, 41 ± 8 µg/day, and 33 ± 7 µg/day. From day 28 through the end of the study, median (IQR) plasma concentrations were 0.22 (0.18–0.27) ng/mL, 0.28 (0.24–0.33) ng/mL, and 0.31 (0.28–0.33) ng/mL for ISL in Groups 1, 2, and 3, respectively, and 0.26 (0.21–0.31) ng/mL, 0.25 (0.16–0.47) ng/mL, and 0.30 (0.23–0.38) ng/mL for ENG in Groups 1, 2 and 3, respectively. Implants across all groups were well tolerated and demonstrated favorable local tolerability over the 90-day dosing period. **Conclusions**: Integration of multiple indications into a single platform capable of sustained release over an extended duration holds potential to address persistent gaps in women’s sexual and reproductive health needs.

## 1. Introduction

Unintended pregnancies and new human immunodeficiency virus (HIV) infections remain significant global public health challenges, with an estimated 121 million unintended pregnancies and 1.3 million new HIV cases reported each year [1,2,3]. These interconnected health concerns disproportionately impact populations in low- and middle-income settings, adversely affecting health outcomes and life expectancy [4,5,6]. In 2018, approximately 1.3 million pregnant women were living with HIV globally, 90% of whom resided in sub-Saharan Africa, where unintended pregnancies remain common among women including women using contraception [7]. Evidence from sociobehavioral research reveals a strong inclination towards long-acting (LA) and reversible preventive options over daily or on-demand regimens, such as oral pills or condoms [8,9,10,11]. To this end, there exists a critical need to develop drug delivery platforms that are safe, effective, user-friendly, and tailored to meet the sexual and reproductive needs of women across different life stages and circumstances.

To date, there has been tremendous progress in the development of LA hormonal contraceptive and HIV pre-exposure prophylaxis (PrEP) modalities. DepoProvera is a hormonal contraceptive injectable administered every 3 months [12], with vaginal rings offering a more user-controlled option that can last up to 1 month (NuvaRing) [13] or 1 year (Annovera) [14]. Even longer durations have been approved in the form of intrauterine devices and implants, including Mirena which is approved for up to 8 years [15], and Kyleena [16], Nexplanon [17], and Jadelle [18] which are approved for up to 5 years. A once-monthly dapivirine vaginal ring for HIV PrEP has been approved in several African countries [19] and received positive opinion from the European Medicines Agency (EMA) [20], but has not been approved in the United States (US) due to the existing landscape of HIV prevention options [21]. Recent US FDA approvals of LA injectables such as cabotegravir [22] (dosed every 2 months) and lenacapavir [23] (dosed every 6 months) have transformed LA HIV PrEP. While both contraceptive and HIV PrEP products may be used independently, synchronizing administration schedules can be challenging, particularly in low- and middle-income countries with limited healthcare access [24].

An integrated approach combining a hormonal contraceptive and an antiretroviral (ARV) would offer a simplified regimen for women seeking dual protection. Despite considerable progress in the multipurpose prevention technology (MPT) product portfolio, on-demand female and male condoms remain the only widely accessible options to date. Platforms enabling dual release of active pharmaceutical ingredients (APIs) face key technical challenges such as coformulation of multiple APIs with different physicochemical properties, potential drug–drug interactions, and integration into drug–device combination products [25]. Moreover, achieving controlled and simultaneous release of therapeutics with divergent pharmacokinetic profiles and mechanisms without compromising drug stability or bioactivity requires tailored delivery systems [26,27,28]. Importantly, offering women a range of effective options that align with their lifestyles is essential to maximize uptake and improve adherence [29]. To address this unmet need and accommodate the diverse sexual and reproductive health priorities for women, researchers have developed MPTs [30] encompassing pills [31,32], injectables [33,34,35], implants [36,37], patches [38], and non-systemic, user-controlled options such as vaginal rings [39,40,41,42], films [43,44], and inserts [45]. Although the Dual Prevention Pill (DPP) is poised to achieve regulatory approval in 2026 as the first MPT since condoms [46], most candidates remain under preclinical or early clinical stages of development.

Herein, we report the design and evaluation of a subcutaneous polymer-based reservoir implant, capable of delivering APIs for extended timeframes, with the added advantage of reversibility to allow a return to fertility or to modify therapeutic regimens. The implant utilizes the slow-degrading poly(ε-caprolactone) (PCL) polymer widely used in controlled drug delivery [47,48,49]. Our previous work demonstrated the release of contraceptive hormone etonogestrel (ENG) and ARV islatravir (ISL) from separate implants in rodents for 6 months [50]. Continued formulation optimization efforts have facilitated the development of a single implant that can simultaneously deliver both an ARV and a hormonal contraceptive. To the best of our knowledge, this is the first reported single-rod MPT implant demonstrating concurrent release of distinct APIs for combination prevention.

## 2. Materials and Methods

### 2.1. Fabrication of Implants

The implant formulations contained either ISL (Sundia MediTech Company, Shanghai, China), or ENG (AdooQ Bioscience, Irvine, CA, USA), mixed with excipients PEG_40_ castor oil and glycerol (Croda International, Snaith, UK). Poly(ε-caprolactone) (PCL) pellets, PC17 (molecular weight M_n_ 67 kDa, Corbion, Amsterdam, The Netherlands), were extruded as hollow cylindrical tubes using a hot-melt, single screw extrusion process at GenX Medical (Chattanooga, TN, USA). To ensure compatibility with market-available trocars, the outer diameters of these tubes were restricted to 2.5 mm. All implants had a wall thickness of 200 µm and contained the same formulation for each API (1:1 *w*/*w*% ratio of ISL: glycerol, 2:1 *w*/*w*% ratio of ENG: PEG_40_ castor oil). Stability studies demonstrated that both APIs remained stable in their respective formulations following incubation at 37 °C for more than 10 days, as evidenced by high API purity (Table S1 in Supplementary Materials). Using a custom heat sealing apparatus, SEALER-01 (Figure S1A,B in Supplementary Materials), PCL tubes were enclosed using molten PCL as previously described [51,52]. ENG segments were designed to have an active reservoir length of 5 mm and the formulations were loaded into the PCL tubes using a modified stainless steel spatula. ISL segments had an active reservoir length of 30 mm and the formulations were loaded using a 3-mL syringe fitted with a 15-gauge blunt tip needle. Once the formulation had filled the reservoir to the required length, any remaining material on the interior tube wall was removed using a stainless steel rod, and a final cleaning was performed with a cotton swab. A 1 mm thick cross-section of a PCL solid rod was placed inside the tube and then sealed at the remaining open-end of the implant with molten PCL using SEALER-01. To account for slight differences in implant dimensions, drug reservoir area was measured with ImageJ (Version 1.50e, NIH, Bethesda, MD, USA), and release rates were normalized to the surface area of the implant. The intercompartmental PCL spacer or seals on either end of the implants were not included in calculations of the implant surface area.

Group 1 fabrication: Two separate PCL tubes were joined by a single PCL spacer using a benchtop heat sealing apparatus designed and manufactured by Gilero, A Sanner Group Company (Durham, NC, USA), SEALER-02 (Figure S1C in Supplementary Materials). Solid PCL rods, cut into 5 mm sections, were positioned inside two empty PCL tubes so that the tube ends were in contact and the PCL spacer was centered evenly between them. Stainless steel rods were inserted into each tube for support, and the entire assembly was gently placed in SEALER-02. The stainless steel rods additionally served to center the seal within the device, ensuring both tubes were aligned evenly. The jaws of SEALER-02 were heated to 58.5 °C and upon activation with a foot pedal, they were programmed to hold the assembly together for 30 s at a pneumatic pressure of 25 psi. After 30 s, the jaws disengaged from the assembly. To provide stability to the center PCL spacer, it was best practice to hold the tubes down on either side of the jaws during this step. The heat-sealed assembly was then cooled to room temperature for approximately 30 s prior to removing the entire assembly from SEALER-02 (Figure S2). Once the segmented implants were fabricated, ISL segments were loaded with formulation and sealed followed by the formulation loading and sealing of the ENG segments with SEALER-01.

Group 2 fabrication: A PCL spacer was placed in a single PCL tube and heat-sealed using SEALER-02 following the protocol detailed in the section above (Figure S3 in Supplementary Materials). The PCL spacer separated the PCL tube into two compartments that were then loaded with ISL formulation in one segment and ENG formulation in the other.

Group 3 fabrication: To fabricate the individual implants that served as controls, previously established protocols were followed [51,52]. Briefly, PCL tubes were trimmed to the correct length to achieve an implant with the desired reservoir length (5 mm for ENG or 30 mm for ISL) with 3 mm headspace at each end for sealing. PCL tubes were sealed on one end with molten PCL using SEALER-01 [51]. After the formulation was loaded and the interior of the tube was cleaned, the implant was sealed in a similar manner to the first seal.

### 2.2. Implant Sterilization

All implants were fabricated under aseptic conditions using a biosafety cabinet and irradiated with a dose range of 18–24 kGy at room temperature, using a Cobalt-60 gamma-ray source (Nordion Inc., Ottawa, ON, Canada) at Steris (Libertyville, IL, USA).

### 2.3. In Vitro Release Testing

API-loaded implants were placed in sterilized polypropylene tubes filled with 1× phosphate-buffered saline (PBS, pH 7.4) at physiological temperature (37 °C) with gentle agitation (104 rpm). The implants were transferred to fresh buffer solutions 2×/week in a biosafety cabinet to maintain sink conditions. The concentrations of ENG and ISL released in the PBS buffer were measured on Agilent 1200 HPLC-UV-Vis (Agilent Technologies, Santa Clara, CA, USA) using an Agilent Zorbax SB-C18 (4.6 mm × 50 mm) column (Agilent Technologies, Santa Clara, CA, USA) and used to calculate the daily release rates from the implant.

### 2.4. Mechanical Testing of Segmented Implant Configurations

An MTS electromechanical testing system (MTS Systems Corporation, Eden Prairie, MN, USA) equipped with a calibrated load-cell of 5000 N was used to conduct tensile testing of all implant configurations. Implants were tested without active or placebo formulations to isolate the contribution of the central seal to mechanical integrity; thus, Group 1 and Group 2 samples were fabricated only with the PCL rod spacer and were not sealed at the terminal ends. Each tube was aligned within the grips, clamped with a gauge length of 20 mm, and elongated at a constant crosshead speed of 200 mm/min until failure. For each configuration, five replicates were tested. Mechanical parameters, including stress–strain behavior, elastic modulus, percent elongation, and ultimate tensile strength (UTS), were calculated using TestWorks^®^ version 4 instrument software (MTS Systems Corporation, Eden Prairie, MN, USA). Non-parametric unpaired *t*-tests were performed to compare data across Groups 1 and 2.

### 2.5. In Vivo Studies in New Zealand White (NZW) Rabbits

All animal studies were conducted in accordance with a protocol approved by the local Institutional Animal Care and Use Committee (IACUC) at the University of Pittsburgh and Magee Womens Research Institute, according to the provisions of the Animal Welfare ACT, PHS Animal Welfare Policy, and the principles of the NIH Guide for the Care and Use of Laboratory Animals.

Female NZW rabbits (2–3 kg) were anesthetized and a small incision in the dorsal scapular region was made. The implants were positioned in the subcutaneous space using a Sino-II trocar and the incision was closed with sterile skin glue. Animals were monitored until they recovered.

The treatment groups (n = 4/group) consisted of Group 1 (a two-compartment implant consisting of an ENG-containing tube joined together with an ISL-containing tube), Group 2 (a single implant consisting of a spacer between the ENG-containing compartment and the ISL-containing compartment), and Group 3 consisting of a single ENG implant and a single ISL implant. The incorporation of contralateral implantation was used for Group 3. Groups 1 and 2 were evaluated through Day 91, whereas Group 3 was evaluated through Day 90; for simplicity, the study duration is referred to as 90 days throughout the manuscript.

No formal a priori sample-size calculation or statistical power analysis was conducted because this was an exploratory/proof-of-concept study. Rabbits were allocated to each group to achieve comparable distributions of baseline body weight across treatment groups. No formal randomisation sequence was generated. Rabbits were singly housed, and cage locations remained fixed throughout the study; cage location was not randomized or rotated across treatment groups. Implantation procedures were completed for all rabbits within one treatment group before proceeding to the next group; therefore, treatment administration order was not interspersed or randomized.

Blinding was not implemented as the primary outcome was quantitative drug-concentration analyses in plasma. The study personnel responsible for group allocation were aware of the assigned treatment groups. Personnel conducting the implantation procedures and subsequent animal monitoring/outcome assessments were aware of group allocation. Data were analyzed by investigators aware of the treatment assignments.

No a priori inclusion or exclusion criteria were established for animals, and all animals that were enrolled and completed the experimental procedures were included in the analyses.

### 2.6. Pharmacokinetic (PK) Analyses

At indicated times points, blood samples were collected and processed to plasma to determine ENG and ISL. The plasma concentrations of ENG and ISL were determined using liquid chromatography with mass spectrometry (LC-MS/MS). Plasma samples were extracted by either liquid–liquid precipitation (ENG) or protein precipitation (ISL) using stable isotopically labeled internal standards (ENG-d_7_, ^13^C^15^N_3_-ISL). Plasma extracts were analyzed by LC-MS/MS with detection on an AB Sciex API-6500+ triple quadrupole mass spectrometer (AB Sciex LLC, Marlborough, MA, USA). The calibration ranges for ENG and ISL in plasma were 0.100–500 ng/mL and 0.025–250 ng/mL, respectively. For ENG samples, the effective LLOQ was sample-specific based on the plasma volume available for analysis; the ISL LLOQ was 0.025 ng/mL. Precision and accuracy of the calibration standards and quality control samples were within 15%.

The maximum serum concentration, *C*_max_, and time of maximum concentration, t_max_, were direct observations from the mean plasma concentration vs. time curve. Exposure of animals to ENG and ISL expressed as area under the plasma concentration-time curve (AUC) from zero to the last measurable sample, AUC_(0t)_, was obtained by means of non-compartmental analysis (NCA) in PKSolver version 2.0. AUC_(0t)_ was calculated by means of the linear up-log down method.

### 2.7. Safety Evaluation

On termination day (Day 91 for Groups 1 and 2; Day 90 for Group 3), macroscopic examination of abnormalities (erythema or inflammation) in tissues surrounding the implant sites were evaluated by a board-certified veterinarian.

### 2.8. Statistical Analyses

Plasma concentration–time data are presented as the median and interquartile range (IQR) of individual animal concentrations at each sampling time point. Summary plasma concentrations from Day 28 through the end of the study were calculated as the median (IQR) of pooled individual animal concentrations across all sampling time points within this interval. Concentrations below the lower limit of quantification (LLOQ) were assigned a value of one-half the corresponding LLOQ for graphical presentation and calculation of descriptive summary statistics [53]. Descriptive statistics and graphical analyses were performed using GraphPad Prism version 10.1.2 (GraphPad Software, Boston, MA, USA).

### 2.9. Assessment of Excised Implants

After removal of implants from rabbits at the terminal time point, the purities of the residual APIs remaining within the implant core were analyzed using an Agilent 1260 Infinity II HPLC (Agilent Technologies, Santa Clara, CA, USA) equipped with a Zorbax Bonus-RP, 4.6 mm × 150 mm, 3.5 µm column (Agilent Technologies, Santa Clara, CA, USA). The ISL or ENG segment was fully submerged in tetrahydrofuran (THF) and allowed to dissolve overnight. The resultant THF solutions containing the entirety of the residual API were diluted 250-fold for ENG and 5000-fold for ISL with 1:1 acetonitrile: water *v*/*v*% and the purities of ENG or ISL were calculated as the percent of the peak area associated with the API relative to the total peak area in the HPLC spectra.

## 3. Results and Discussion

### 3.1. Design and Fabrication of Single-Rod, Segmented Implant Configurations

**Figure 1:**
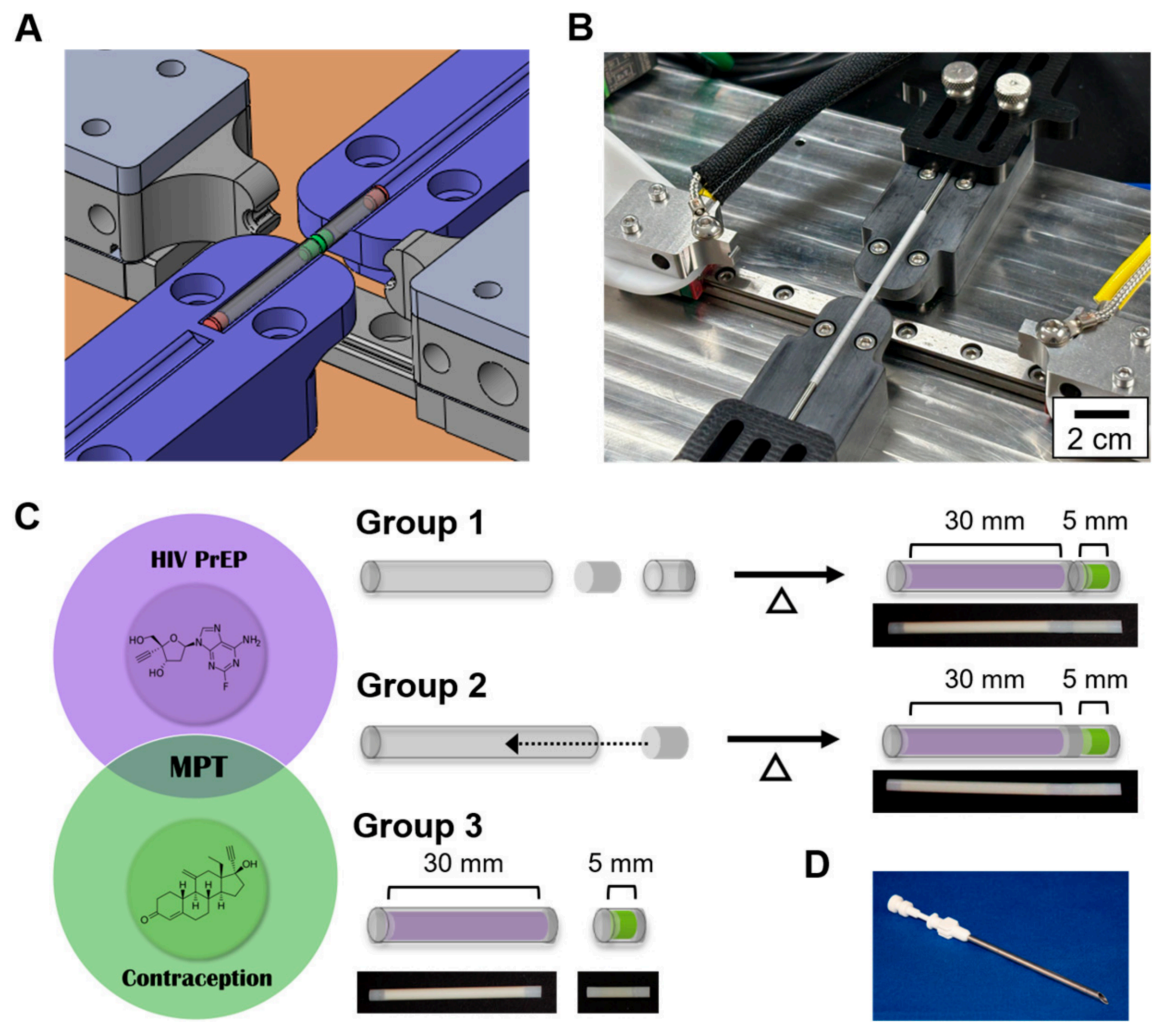
(**A**) A SolidWorks 2018 schematic of SEALER-02. (**B**) Digital image of SEALER-02 with an implant positioned in the sealing groove. (**C**) Schematic of fabrication of Group 1 (separate PCL tubes joined via a PCL rod spacer), Group 2 (single PCL tube segmented by inserting a PCL rod spacer; dashed arrow indicates the direction of PCL spacer insertion into the PCL tube), and Group 3 (individual ISL and ENG) MPT implants for dual prevention of HIV and pregnancy. (**D**) Digital image of Sino II trocar used to subcutaneously administer implants.

We previously reported on separate ISL- and ENG-containing implants that were administered at distinct sites in female Wistar rats using the Sino-II trocar [50]. To simplify administration and enhance user convenience, we sought to engineer a trocar-compatible, single-rod implant configuration capable of concurrent dual-API delivery from a single implantation site. Moreover, by incorporating separate segmented compartments for each API, we could independently tailor release characteristics and prevent potential drug–drug interactions at the formulation level. The fabrication was enabled by SEALER-02, a custom apparatus designed to produce single-rod implants with discrete compartments for independent formulation loading (Figure 1A,B and Figures S1–S3 in Supplementary Materials).

We developed two single-rod configurations, both utilizing a short PCL rod spacer (5 mm) positioned between the ISL and ENG compartments to create discrete compartments for the individual drug formulations (Figure 1C). The first approach (Group 1) involved joining two separate PCL tubes with the spacer placed between them, followed by heat-sealing to produce an integrated implant. The second configuration (Group 2) employed a single PCL tube that was partitioned by inserting the spacer between two drug-containing compartments, followed by heat-sealing to form isolated drug reservoirs. Both fabrication strategies yielded functional single-rod implants capable of concurrent dual-API delivery, with Groups 1 and 2 having identical overall implant dimensions and equivalent lengths of the respective drug-containing compartments. In addition, we fabricated individual ISL- and ENG-containing implants that were co-administered as a set to provide dual prevention (Group 3), serving as a control to evaluate whether the fabrication of single-rod configurations in Groups 1 and 2 altered release behavior relative to separate implants.

Single-rod configurations could offer key advantages over separate implants, including simplified administration via a single trocar insertion (Figure 1D), reduced implantation time, and lower procedural complexity, which are critical factors for scalability and user acceptability in resource-limited settings [54]. By consolidating dual protection into one implant, these designs could also minimize patient burden and enhance adherence potential for MPTs [1]. Furthermore, the biodegradable PCL design could eliminate the need for a clinic visit for implant removal following drug depletion, further reducing patient and healthcare burden.

### 3.2. Optimization of API Formulation and Implant Length

Previously, we developed MPT implants comprising a set of separate ISL and ENG implants that were inserted at separate sites to deliver APIs at sustained, targeted protective levels [50,55]. In our previous implant designs, the ENG implants (300 µm wall thickness) remained structurally intact and were readily retrieved, whereas the ISL implants (100 µm wall thickness) exhibited compromised mechanical integrity. Here, transitioning to a thicker wall for the ISL segment would support long-term structural robustness and facilitate ease of retrieval. To streamline manufacturing of segmented implants, we sought to standardize wall thickness across both ISL and ENG segments, while maintaining an overall implant length of ≤46 mm for compatibility with commercial trocars. This overall length is comparable to clinically used contraceptive implants, such as Nexplanon (40 mm length × 2 mm diameter) [56] and Sino-implant (II) (44 mm length × 2.4 mm diameter) [57]. Accordingly, subsequent efforts to develop single-rod MPT implants were focused on maintaining a single wall thickness of 200 µm for the entire two-compartment implant, while evaluating alternate API formulations to ensure sustained release profiles at protective levels.

Prior investigations have established that the drug release rate can be modulated by varying the wall thickness, implant length, and excipient choice, thereby allowing for tunable and sustained drug delivery profiles [51]. ISL formulated with sesame oil and loaded within 100 µm wall thickness implants exhibited sustained release at approximately 70 µg/day [50]. As expected from the inverse relationship between wall thickness and release rate [51], the same formulation loaded into implants with a 200 µm wall thickness yielded a reduced release rate of 13.9 ± 2.7 µg/day over 90 days (Figure S4 in Supplementary Materials). To achieve higher drug release rates, formulation optimization efforts included the use of release-enhancing excipients. Although most evaluated excipients formulated with ISL did not exhibit zero-order release profiles, glycerol was identified as the lead excipient, delivering sustained, near zero-order kinetics that enabled direct comparison across the single-rod, segmented implant configurations. The ISL segment length was limited to 30 mm to maximize the ISL release rate while leaving sufficient space within the overall device for the intercompartmental PCL spacer, terminal seals, and the ENG segment.

Given the 46 mm maximum implant length for trocar compatibility, and the central and terminal seals requiring 11 mm, only 5 mm was available for the ENG segment. Optimization steps for ENG included reducing the wall thickness from 300 µm to 200 µm to increase release rates [51], as well as using PEG_40_ castor oil as an excipient to improve drug solubility and achieve higher release rates suitable for sustained delivery.

Evidence of API chemical stability during prolonged implant release was obtained from 6-month in vitro studies conducted at 37 °C. Analysis of residual formulations recovered from the implants at the end of the study demonstrated that ENG and ISL retained approximately 99% and 96% chemical purity, respectively (Table S1 in Supplementary Materials). These findings support the chemical stability of both APIs within their respective implant formulations during prolonged in vitro release at physiological temperature. However, formal real-time and accelerated storage-stability studies of the finished implant product will be required to establish appropriate storage conditions and product shelf life.

### 3.3. Tensile Testing of Single-Rod Implant Configurations

**Figure 2:**
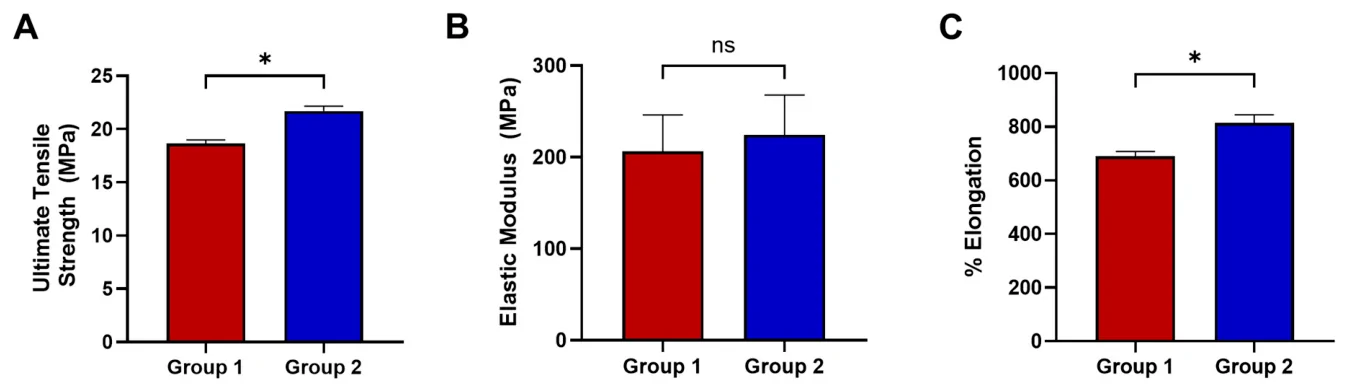
Mechanical properties of empty segmented implants from Groups 1 and 2 demonstrating (**A**) ultimate tensile strength, (**B**) elastic modulus, and (**C**) percent elongation at failure (n = 5 independent samples per group; * *p* value = 0.0159 for UTS and % elongation; ns: non-significant for elastic modulus).

Tensile testing was performed to confirm mechanical robustness of the intercompartmental seals and to identify potential differences arising from the two implant fabrication approaches for Group 1 and Group 2. To isolate the effects of implant architecture and fabrication method, rather than formulation-dependent effects, tensile testing was conducted using empty implants. Because tensile testing requires elongation of the implant to failure, no API or surrogate material was loaded into the implants to avoid the release of the encapsulated material during testing. When comparing both implant configurations, Group 2 demonstrated slightly higher mechanical strength than Group 1. The ultimate tensile strength (UTS) was 18.6 ± 0.4 MPa for Group 1 and 21.7 ± 0.5 MPa for Group 2, indicating that the latter group tolerated higher loads before failure (Figure 2A). Similar stiffness was observed across both groups, with elastic moduli of 206.3 ± 39.8 MPa and 224.3 ± 43.6 MPa for Groups 1 and 2, respectively (Figure 2B). As both configurations were fabricated using the same polymer and wall thickness, differences in elastic modulus were not expected [58]. Group 2 also exhibited higher percent elongation at failure (813.7 ± 30.8%) compared to Group 1 (689.5 ± 18.2%) (Figure 2C). This difference is likely reflective of the fabrication approaches, wherein Group 1 implants were formed by joining separate tubes via a PCL rod spacer and could undergo earlier failure than the continuous single-tube construction of Group 2.

In alignment with the UTS and % elongation data, qualitative assessment of failure modes also revealed distinct behaviors for Group 1 and Group 2 implant configurations. In Group 1, where two separate tubes were joined via a PCL rod spacer, application of tensile force typically caused one tube segment to slip off the intercompartmental seal, resulting in a clean separation while the seal itself remained intact (Figures S5A and S6 for individual stress–strain curves in Supplementary Materials). In contrast, Group 2 implants, fabricated from a single continuous tube with a center spacer, failed by fracture within one of the tube segments rather than at the center seal, producing visibly ragged fracture surfaces (Figures S5B and S7 for individual stress–strain curves in Supplementary Materials). Importantly, the intercompartmental seal remained intact across both fabrication approaches, confirming its structural integrity. Although Group 2 implants exhibited slightly better mechanical performance, likely attributable to the fabrication approach, such extreme tensile loading conditions are unlikely to occur in clinical use. Importantly, the mechanical testing to failure was used to define material limits and performance characteristics, rather than to predict clinical failure under normal use, which would not reach these tensile forces under typical conditions. Consequently, both configurations were evaluated in in vitro and preclinical studies.

### 3.4. In Vitro Performance of Implant Configurations in Parallel to Preclinical Study

**Figure 3:**
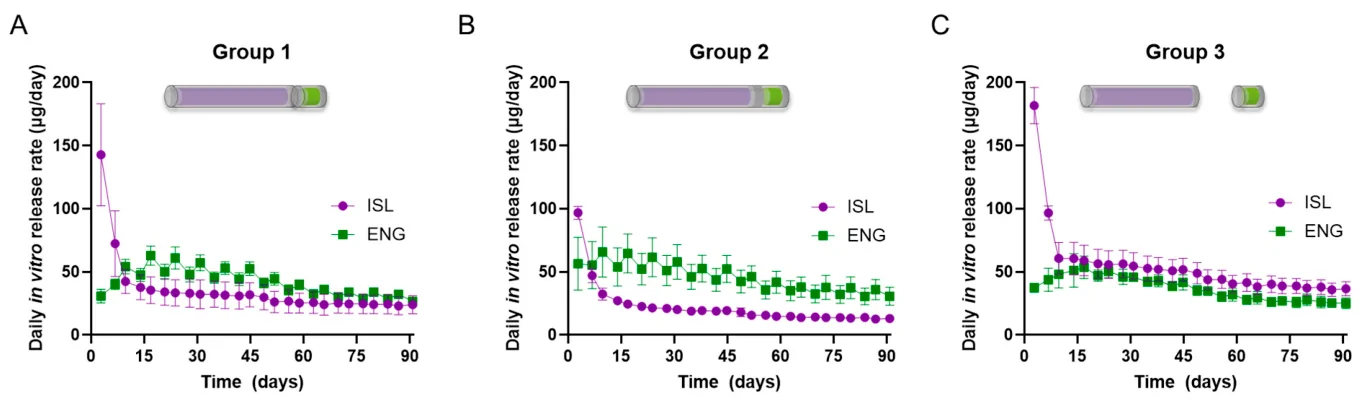
Daily in vitro release profiles of ISL and ENG formulations from implants evaluated in parallel with the 90-day preclinical study for (**A**) Group 1, (**B**) Group 2, and (**C**) Group 3. All implants comprised PC17 polymer with 200 µm wall thickness (ISL segments: 30 mm length; ENG segments: 5 mm length). Profiles represent the mean (standard deviation) of 5 implants at each timepoint.

In parallel with the 90-day preclinical study, in vitro studies were conducted in PBS at 37 °C under controlled sink conditions. While this simplified release medium does not reproduce the biochemical complexity of the subcutaneous environment, previous studies using comparable PBS release conditions have demonstrated close agreement between in vitro and in vivo drug absorption [59]. As shown in Figure 3, an initial increase in release rate was observed for both APIs, likely reflecting progressive hydration of the hydrophilic excipients and the associated enhancement in drug solubility and diffusion [60], but this transient effect resolved within approximately 2 weeks, after which release stabilized for the remainder of the study. For ENG, which was formulated with PEG_40_ castor oil (2:1 *w*/*w*%), the daily in vitro release rates from Day 28–91 for Groups 1, 2, and 3 were 39 ± 9 µg/day, 41 ± 8 µg/day, and 33 ± 7 µg/day, respectively (for individual release profiles, see Figure S8 in Supplementary Materials). For ISL, which was formulated with glycerol (1:1 *w*/*w*%), the daily in vitro release rates from Day 28–91 for Groups 1, 2, and 3 were 27 ± 3 µg/day, 16 ± 3 µg/day, and 44 ± 7 µg/day, respectively (for individual release profiles, see Figure S9 in Supplementary Materials). Possible contributions to the observed variability in ISL release could include differences in membrane wall thickness, seal geometry, and the distribution and physical state of the ISL/excipient mixture within the reservoir. Future studies with larger sample sizes and tighter control of these manufacturing parameters will be needed to better characterize and improve ISL release reproducibility.

At the end of the study, the chemical purity of residual ISL and ENG recovered from implants was assessed to evaluate API stability within their respective formulations. ISL and ENG maintained high chemical purity after exposure to in vitro conditions across all implant groups (Table S2 in Supplementary Materials). Because the segmented architecture was designed to maintain the two formulations in physically independent reservoirs, we additionally evaluated potential intercompartment API transport to verify the integrity of the PCL rod spacer. In both directions, the API from the opposite compartment was undetectable. These findings showed the absence of measurable intercompartment API transport and confirmed that the sealing strategy effectively preserved spatial separation of the formulations.

### 3.5. Evaluation of Safety and PK of Implant Configurations in NZW Rabbits

**Figure 4:**
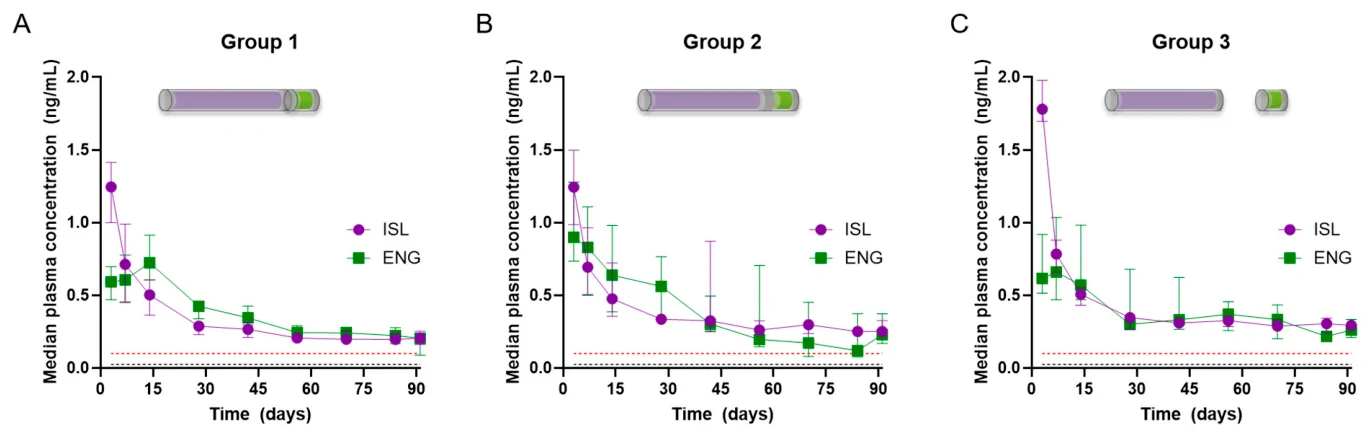
Median (IQR) plasma PK profiles of ISL and ENG over 90 days in NZW rabbits from (**A**) Group 1, (**B**) Group 2, and (**C**) Group 3 MPT implants. Of all ENG plasma measurements across sampling time points, three were below their respective effective LLOQs (0.10–0.13 ng/mL). Concentrations below the LLOQ were assigned a value of one-half the LLOQ for graphical presentation and calculation of summary statistics. The highest effective ENG LLOQ (0.13 ng/mL) and the ISL assay LLOQ (0.025 ng/mL) are indicated by horizontal red and black dotted lines.

Implant configurations were evaluated in female NZW rabbits over a 90-day in vivo study. For Groups 1 and 2, each single-rod MPT implant was inserted subcutaneously using a Sino II trocar. In Group 3, which comprised separate ISL and ENG implants, two separate trocars were used for contralateral placement in each animal. Although a limitation of the present study was the absence of a blank PCL implant control, previous preclinical evaluations of this PCL reservoir-style implant platform have demonstrated favorable local tolerability following subcutaneous administration [50,55,61]. Consistent with the in vitro release profiles (Figure 3), all formulations demonstrated sustained drug release over the study duration. Median plasma ENG concentrations from Day 28 through the end of the study were 0.26 ng/mL (IQR, 0.21–0.31 ng/mL), 0.25 ng/mL (IQR, 0.16–0.47 ng/mL), and 0.30 ng/mL (IQR, 0.23–0.38 ng/mL) for Groups 1, 2, and 3, respectively (Figure 4; for individual profiles, see Figure S10 in Supplementary Materials), which are levels within the established efficacious range for contraception [56]. However, this study was not designed to predict human PK and future work would be required to use translational PK modeling to estimate human ENG exposures from the rabbit data and compare predicted concentrations with these established contraceptive thresholds. Plasma ENG concentrations were generally comparable among the implant groups. Plasma ISL concentrations followed a similar sustained pattern from Day 28 through the end of the study, with median values of 0.22 ng/mL (IQR, 0.18–0.27 ng/mL), 0.28 ng/mL (IQR, 0.24–0.33 ng/mL), and 0.31 ng/mL (IQR, 0.28–0.33 ng/mL) for Groups 1, 2, and 3, respectively (Figure 4; for individual profiles see Figure S11 in Supplementary Materials). One anomalous value was observed for rabbit G7 on Day 42 (1.05 ng/mL ISL), which exceeded the upper outlier threshold defined by the 1.5 × IQR rule (Q3 + 1.5 × IQR = 0.415 ng/mL). This data point was retained in the dataset to demonstrate inter-animal variability. Noncompartmental analysis (NCA) was performed on plasma concentrations of ENG and ISL segments across all implant configurations. The median ± SD *C*_max_ values for ENG were 0.76 ± 0.17, 0.89 ± 0.29, and 0.66 ± 0.28 ng/mL for Groups 1, 2, and 3, respectively, through Day 91 (Table S3 in Supplementary Materials). Given the tight daily dose range (33.2–40.9 µg/day), no statistically significant differences in ENG plasma exposure for *C*_max_ or *AUC*_0–t_ were observed across groups. Corresponding ISL *C*_max_ values were 1.25 ± 0.22, 1.27 ± 0.28, and 1.78 ± 0.15 ng/mL for the same groups, with Group 3 statistically significantly different from Groups 1 and 2 (*p* = 0.007 using the Holm–Sidak pairwise method; Table S4 in Supplementary Materials).

Due to the markedly lower ISL-triphosphate formation in rabbit peripheral blood mononuclear cells (PBMCs) relative to human cells [62], blood samples were not analyzed for intracellular PBMC concentrations. Using a published ISL intravenous PK model for rabbits [63] we deconvolved the plasma concentrations measured in this study to estimate implant absorption rates over our 90-day duration. Across all formulation groups, we estimate that the ISL absorption rate ranged from 11–27 µg/day at 2 weeks post implantation, 7–13 µg/day at 1 month, and 5–12 µg/day at 3 months. These estimated absorption rates are approximately 10-fold below the 100 µg/day oral dosing level that a recent PK/PD modeling study predicts would be effective PrEP [64]. Given this difference, the absorption rates yielded by our system are unlikely to result in effective ISL-triphosphate concentrations within PBMCs. Moreover, a recent macaque efficacy study demonstrated protection against vaginal SHIV challenge at ISL plasma concentrations of ~1.4 ng/mL [61,65], at least 4.5-fold higher than the levels in the present study, suggesting that the concentrations reported here are unlikely to reach the minimum protective threshold [66]. To improve ISL release, formulation optimization could include excipient modification, increasing the ISL segment length, and evaluating alternative polymers that may provide higher overall release. Increasing the ISL segment length and corresponding surface area or reducing wall thickness could enhance drug release, as previously established [51]. However, increases in segment length are constrained by the 45 mm overall implant length required for compatibility with a commercial trocar, expansion of the ISL-containing segment would be possible only within a confined range. Modification of polymer properties, including evaluation of polymers with lower crystallinity and greater molecular diffusivity, may provide an alternative strategy to enhance ISL transport. Nevertheless, the primary objective was achieved for the present study by demonstrating novel implant architectures and manufacturing approaches for single-rod, dual-API delivery. The absence of statistically significant differences in plasma ENG and ISL concentrations across all study groups indicates that drug release rates were independent of implant configuration. Taken together, these findings indicate that the single-rod MPT implants (Groups 1 and 2) achieved sustained systemic delivery of both APIs, with PK performance comparable to the implant design that housed individual APIs (Group 3).

At study termination, all implants were retrieved from the animals with relative ease without any signs of breakage or loss of structural integrity, as expected given the slow degradation profile of PCL. Minimal fibrotic tissue was observed, with no visible evidence of inflammation or erythema in the implant capsule or surrounding tissue (Table S5 in Supplementary Materials). These findings, together with the longitudinal veterinary assessments, support favorable local tolerability of all implant types throughout the study. However, histopathological analysis and quantitative assessment of inflammatory biomarkers were not performed, limiting a more comprehensive characterization of the local tissue response.

Additionally, we assessed the chemical purity of residual ISL and ENG recovered from excised implants and compared these values to those from in vitro studies. Consistent with the in vitro results, both ISL and ENG segments retained their purities across all implant groups (Table S2 in Supplementary Materials).

This study has several limitations. First, further formulation and implant optimization is required to ensure that the ISL segment maintains therapeutic concentrations over the intended delivery interval, as our current design prioritized achieving stable release profiles across groups rather than fully optimized ISL dosing. Second, the in vivo evaluation was limited to 3 months; longer-term studies are needed to more accurately define the duration of effective protection and confirm maintenance of therapeutic levels over time. Third, although PCL is a slow biodegradable polymer with a degradation timeframe often exceeding two years [67], the current study duration was insufficient to characterize changes in molecular weight or fully delineate degradation kinetics for these configurations. Fourth, the in vivo safety evaluation relied primarily on macroscopic and veterinary observations; histopathological analysis of the tissue surrounding the implant and assessment of fibrosis and inflammatory biomarkers were not performed. Future studies incorporating these analyses will be necessary to more comprehensively characterize the local tissue response and biocompatibility of the implants. Despite these limitations, this study outlined advancements in the development of a dual-API implant configured to house independent API formulations, supporting co-delivery of drug with different physiochemical properties.

## 4. Conclusions

This work describes the development and preclinical evaluation of single-rod MPT implants designed to provide simultaneous prevention of unintended pregnancy and HIV. The system was engineered for removability, allowing reversibility to support return to fertility or allow adjustment of therapeutic regimens as needed. The sustained in vitro and in vivo co-delivery of ISL and ENG from segmented single-rod implants highlights the potential of this platform to address a critical gap in the MPT landscape. Segmentation within a single rod may also simplify clinical administration and enhance end-user acceptability by reducing the total number of implants required to achieve dual prevention. To facilitate standardized implant fabrication and align with future scalable manufacture, we developed a customized sealing apparatus to produce single-rod implants with discrete compartments to house independent API formulations. To further develop this fabrication approach, the intercompartmental spacer and terminal seals could be implemented as plugs with standardized dimensions to reduce operator-dependent variability and improve reproducibility of sealing. Although ISL release from the single-rod implants did not achieve the projected targets for HIV PrEP, these results suggest that further optimization could focus on increasing the drug-compartment length, identifying release-modifying excipients, and evaluating membrane properties to enhance drug delivery. In summary, we demonstrate that single-rod MPT implants can deliver an ARV and a hormonal contraceptive at sustained systemic levels. While additional formulation and implant refinement is warranted, the present findings support continued development of PCL-based single-rod MPT implants as a promising LA strategy for dual prevention of HIV and unintended pregnancy in women.

## Data Availability

The datasets generated and/or analyzed during the current study are available from the corresponding author upon reasonable request.

## Supplementary Materials

The following supporting information can be downloaded at: https://www.mdpi.com/article/10.3390/pharmaceutics18091060/s1. Table S1. Chemical purity of ENG and ISL following short-term formulation compatibility testing and long-term in vitro implant release (n = 3). Figure S1. Photographs of SEALER-01 used to make the (A) first end seal, (B) the second end seal, and SEALER-02 to make the (C) intercompartmental seal. Figure S2. Steps to make Group 1 implants with separate PCL tubes joined by a PCL rod spacer. Figure S3. Steps to make Group 2 implants where two independent reservoirs are created within a single PCL tube by a PCL rod spacer. Figure S4. In vitro release profiles of PC17 implants with a wall thickness of 200 µm, 30 mm in length, and loaded with an ISL: sesame oil (1:1 *w*/*w*%) formulation (n = 3 implants). Data are presented as mean ± SD. Figure S5. Digital camera images of empty tubes after tensile testing, in which tubes were purposely elongating to failure to test mechanical properties. Post-tensile testing images shows (A) failure of one segment at the intercompartmental seal for Group 1 implants versus (B) fracture along the tube length for Group 2 implants. Figure S6. Stress-strain curves of Group 1 implants subjected to tensile testing (n = 5 specimens). Figure S7. Stress-strain curves of Group 2 implants subjected to tensile testing (n = 5 specimens). Figure S8. Individual in vitro release profiles of ENG from implants evaluated in parallel with the 90-day preclinical study for (A) Group 1, (B) Group 2, and (C) Group 3 implant configurations (mean shown as red line, n = 5 implants/group). Figure S9. Individual in vitro release profiles of ISL from implants evaluated in parallel with the 90-day preclinical study for (A) Group 1, (B) Group 2, and (C) Group 3 implant configurations (mean shown as red line, n = 5 implants/group). Table S2. Chemical purities of residual ISL and ENG in implants following completion of the in parallel in vitro release study and the 90-day in vivo study. Figure S10. Individual ENG plasma PK profiles in NZW rabbits implanted with Groups 1, 2, and 3 MPT implants over the entire study duration (median shown as red line, n = 4 implants/group). Of all ENG plasma measurements across sampling time points, three were below their respective effective LLOQs (0.10–0.13 ng/mL). The highest effective ENG LLOQ (0.13 ng/mL) is indicated by horizontal dotted lines. Figure S11. Individual ISL plasma PK profiles in NZW rabbits implanted with Groups 1, 2, and 3 MPT implants over the entire study duration (median shown as red line, n = 4 implants/group). ISL lower limit of quantification (LLOQ) shown as horizontal dotted line, 0.025 ng/mL. Asterisk (*) in panel B indicates an outlier for rabbit G7 on Day 42 (1.05 ng/mL ISL), exceeding the 1.5 × IQR upper threshold (0.415 ng/mL); included for variability but excluded from statistical analyses. Table S3. PK parameters (median ± SD) calculated from mean plasma concentrations of ENG segments from Day 28–91 in NZW rabbits. Table S4. PK parameters (median ± SD) calculated from mean plasma concentrations of ISL segments from Day 28–91-day study in NZW rabbits. Table S5. Macroscopic evaluation of abnormalities (fibrosis, erythema or inflammation) in tissues surrounding the implant sites in the NZW rabbits at Day 91 as rated as the following: Severity: N = None (0); 1 = Minimal; 2 = Mild; 3 = Moderate; 4 = Marked; 5 = Severe.
