# Supplementary material for: Development and Preclinical Evaluation of a Dual-Drug Implant for Long-Acting HIV Prevention and Contraception
Source: Pharmaceutics. 2026 Aug 26;18(9):1060. doi: 10.3390/pharmaceutics18091060 (PMC13610647; doi:10.3390/pharmaceutics18091060)
Supplement: Supplementary file 1 [file pharmaceutics-18-01060-s001.zip › pharmaceutics-4500499-supplementary.pdf]

Development and Preclinical Evaluation of a Dual-Drug Implant for Long-Acting HIV Prevention and Contraception

Archana Krovi[1], Leanna Levin[1], Greg J. Gatto[1], Ellen H. Luecke[1], Rhonda Brand[2], Amanda Swistok[2], Mackenzie L. Cottrell[3†], Amanda P. Schauer,[3] Leah M. Johnson[1*]

[1]RTI International, 3040 E. Cornwallis Road, Research Triangle Park, Durham, NC 27709, USA
[2]Department of Medicine and Magee-Womens Research Institute, University of Pittsburgh, Pittsburgh, PA 15213, USA
[3]Division of Pharmacotherapy and Experimental Therapeutics, Eshelman School of Pharmacy, University of North Carolina, Chapel Hill, NC 27599, USA
*Corresponding author leahjohnson@rti.org
†Current address: Department of Pharmacy, Clinical & Administrative Sciences, University of Oklahoma College of Pharmacy, Oklahoma City, OK, 73117, USA

Table S1. Chemical purity of ENG and ISL following short-term formulation compatibility testing and long-term *in vitro* implant release (n=3).

Formulation	API purity after 10-day API/excipient incubation at 37°C (%)	API purity recovered from implants after 6-month *in vitro* release at 37°C (%)
2:1 ENG: PEG40 castor oil	98.9%	99.4 ± 0.1
1:1 ISL: glycerol	98.7%	96.3 ± 0.3

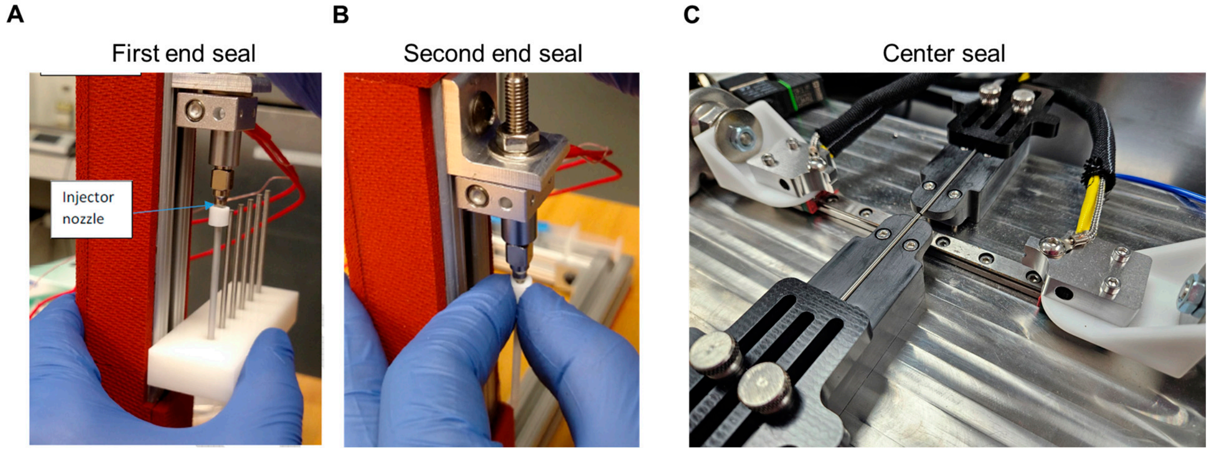

Figure S1. Photographs of SEALER-01 used to make the (A) first end seal, (B) the second end seal, and SEALER-02 to make the (C) intercompartmental seal.

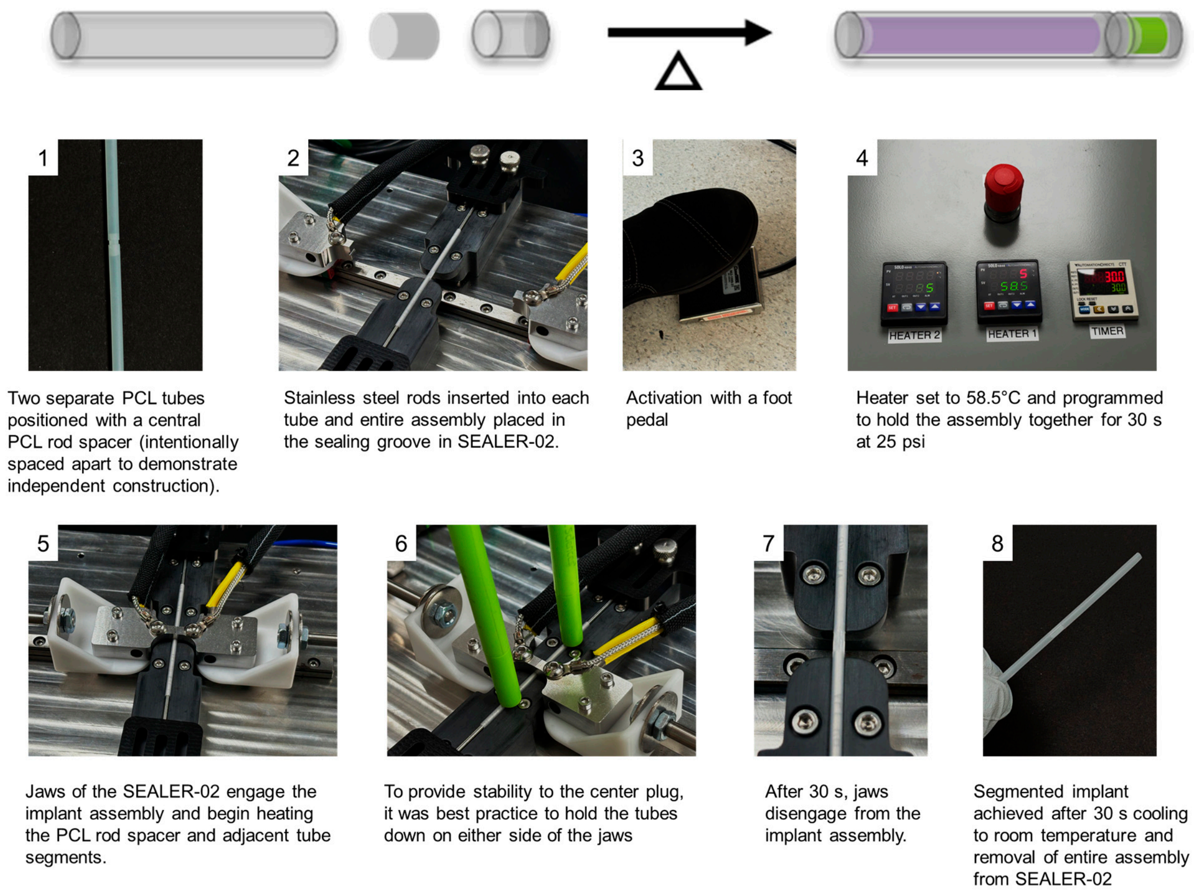

Figure S2. Steps to make Group 1 implants with separate PCL tubes joined by a PCL rod spacer.

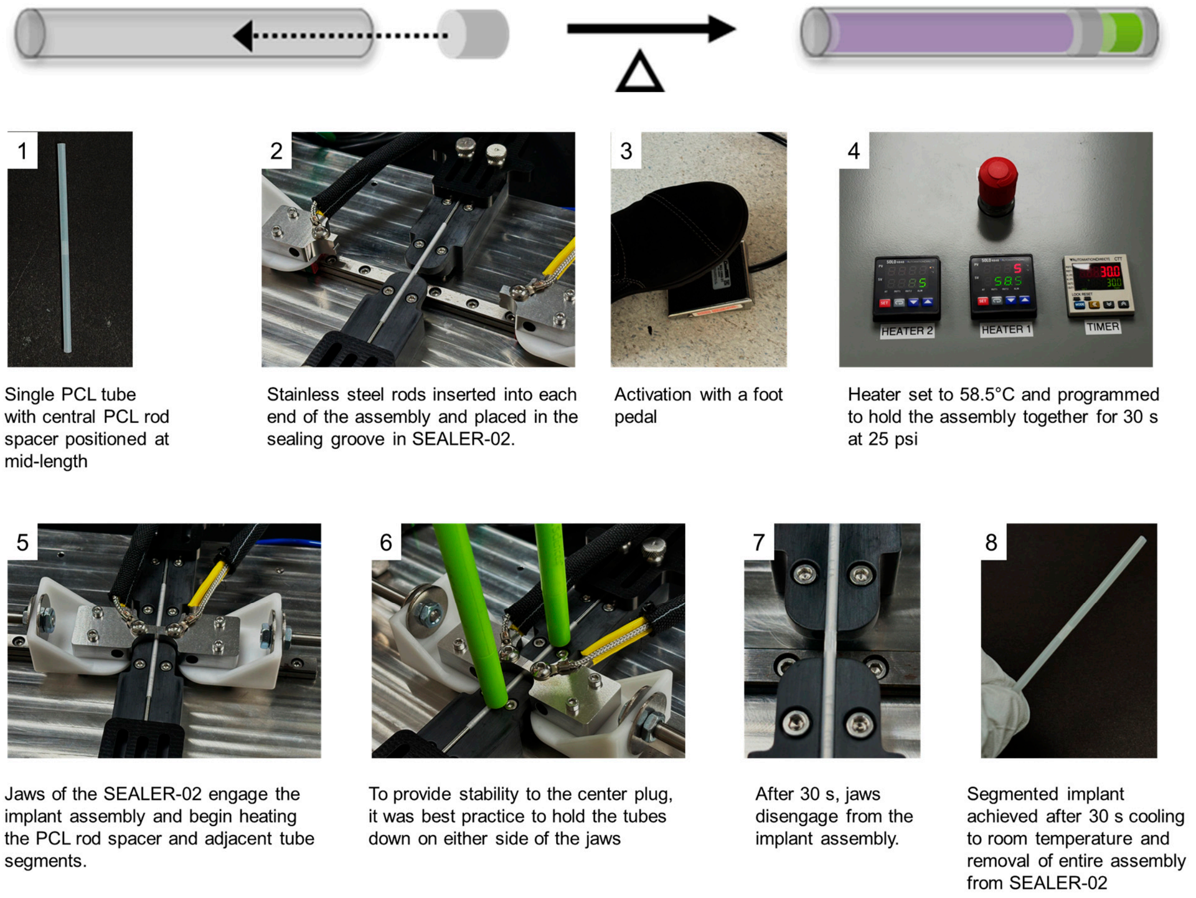

Figure S3. Steps to make Group 2 implants where two independent reservoirs are created within a single PCL tube by a PCL rod spacer.

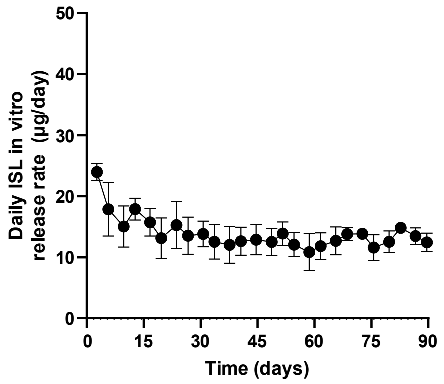

Figure S4. *In vitro* release profiles of PC17 implants with a wall thickness of 200 µm, 30 mm in length, and loaded with an ISL: sesame oil (1:1 w/w%) formulation (n = 3 implants). Data are presented as mean ± SD.

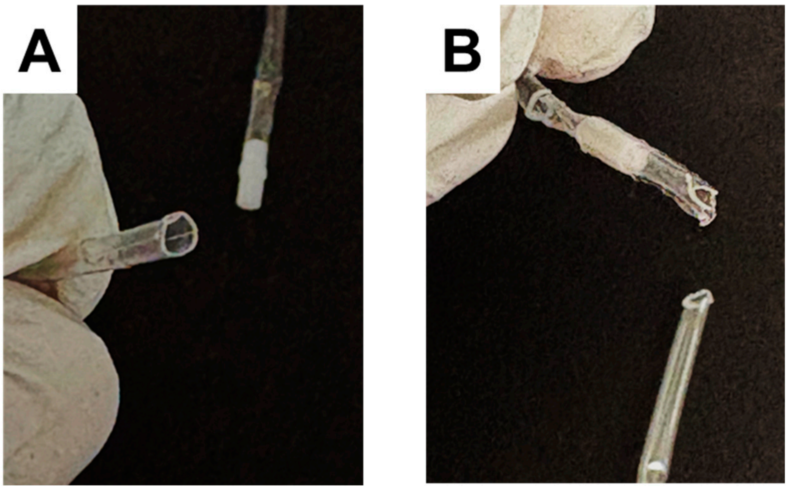

Figure S5. Digital camera images of empty tubes after tensile testing, in which tubes were purposely elongating to failure to test mechanical properties . Post-tensile testing images shows (A) failure of one segment at the intercompartmental seal for Group 1 implants versus (B) fracture along the tube length for Group 2 implants.

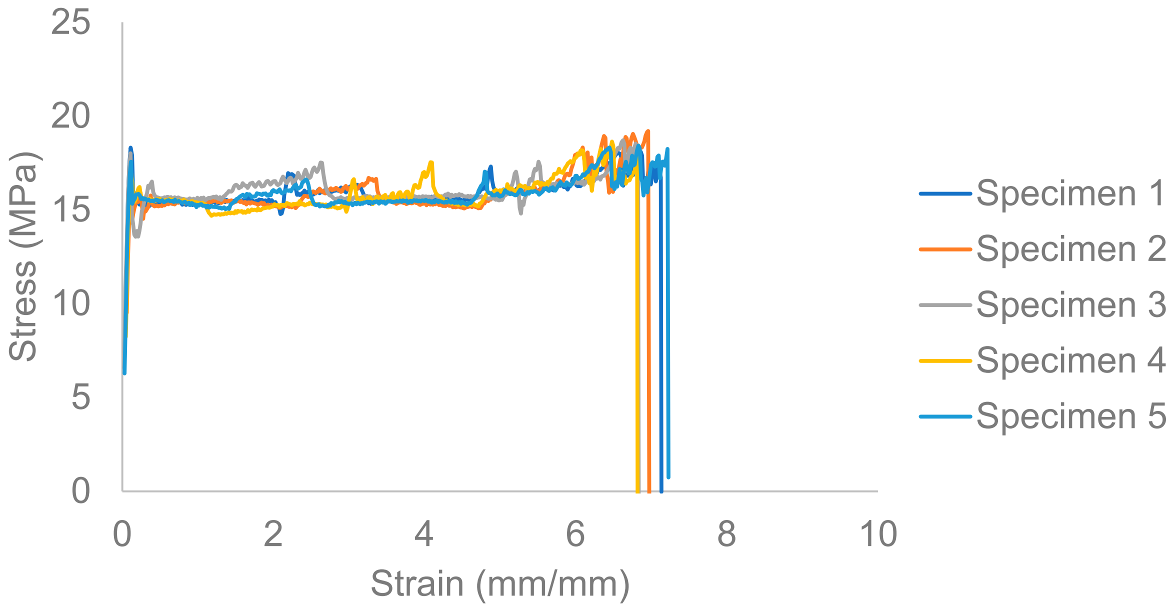

Figure S6. Stress-strain curves of Group 1 implants subjected to tensile testing (n = 5 specimens).

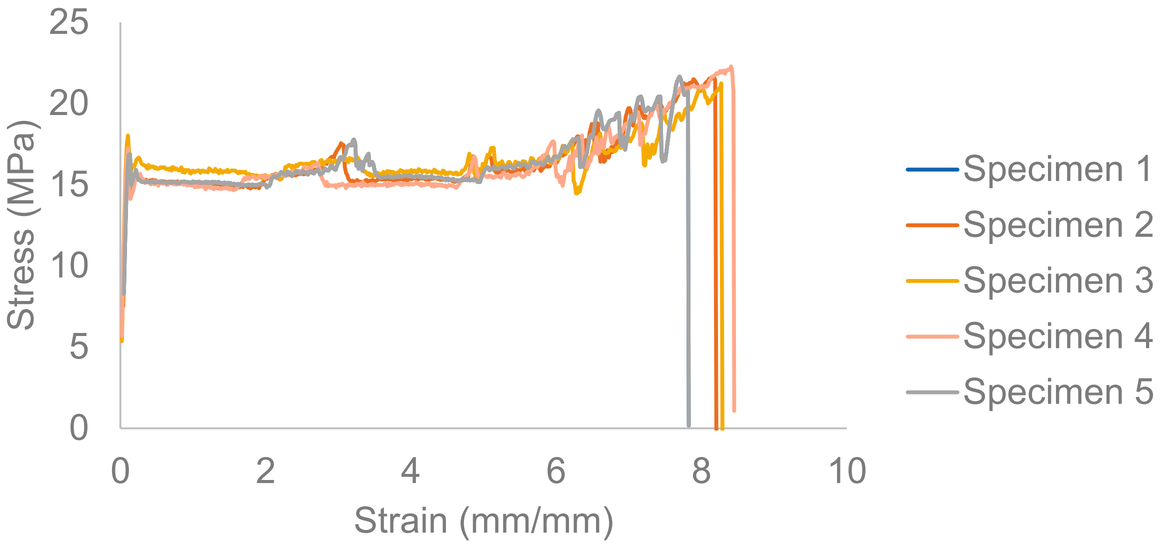

Figure S7. Stress-strain curves of Group 2 implants subjected to tensile testing (n = 5 specimens).

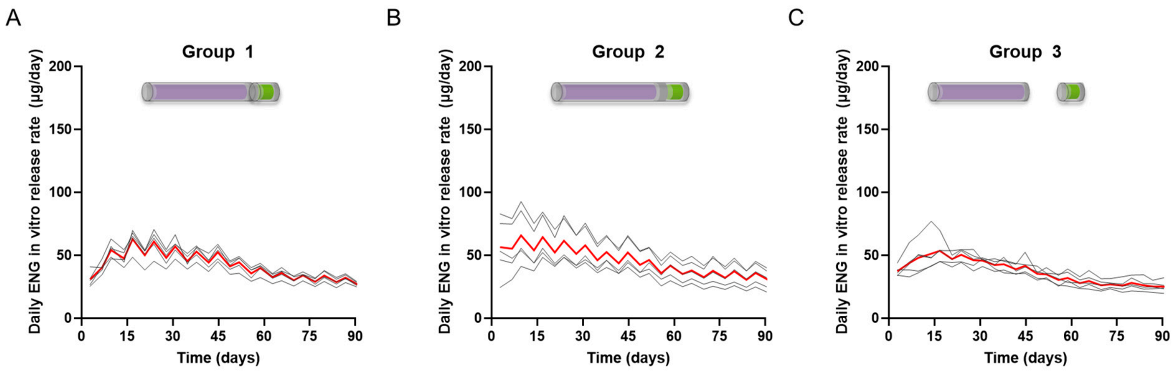

Figure S8. Individual *in vitro* release profiles of ENG from implants evaluated in parallel with the 90-day preclinical study for (A) Group 1, (B) Group 2, and (C) Group 3 implant configurations (mean shown as red line, n = 5 implants/group).

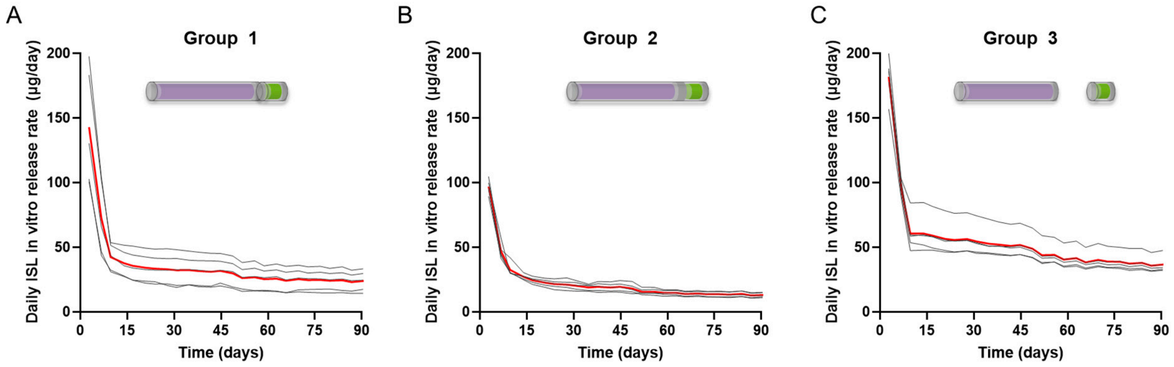

Figure S9. Individual *in vitro* release profiles of ISL from implants evaluated in parallel with the 90-day preclinical study for (A) Group 1, (B) Group 2, and (C) Group 3 implant configurations (mean shown as red line, n = 5 implants/group).

Table S2. Chemical purities of residual ISL and ENG in implants following completion of the in parallel *in vitro* release study and the 90-day *in vivo* study.

Group	Remaining ENG chemical purity (%)		Remaining ISL chemical purity (%)	
	In vitro	In vivo	In vitro	In vivo
1	97.2 ± 0.4	96.8 ± 0.2	99.3 ± 0.1	99.4 ± 0.1
2	98.8 ± 0.1	96.5 ± 0.3	99.3 ± 0.1	99.5 ± 0.1
3	98.1 ± 0.2	97.5 ± 0.1	99.2 ± 0.1	99.4 ± 0.1

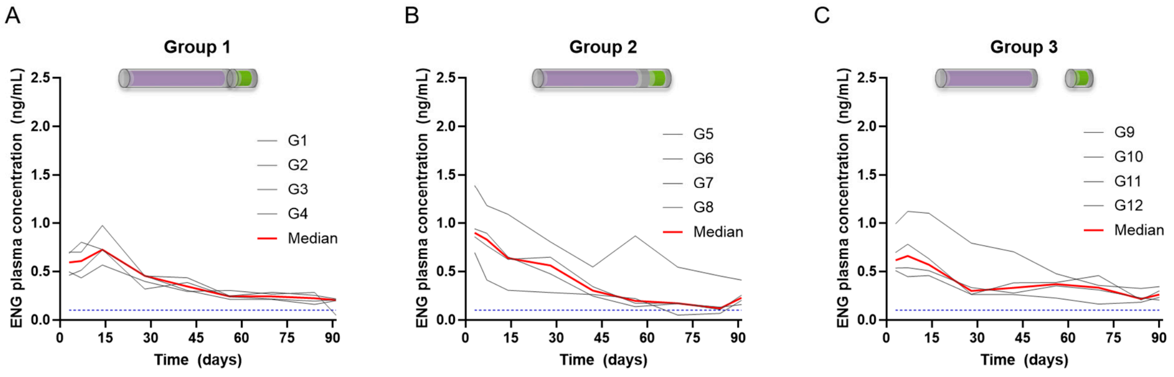

Figure S10. Individual ENG plasma PK profiles in NZW rabbits implanted with Groups 1, 2, and 3 MPT implants over the entire study duration (median shown as red line, n = 4 implants/group). Of all ENG plasma measurements across sampling time points, three were below their respective effective LLOQs (0.10–0.13 ng/mL). The highest effective ENG LLOQ (0.13 ng/mL) is indicated by horizontal dotted lines

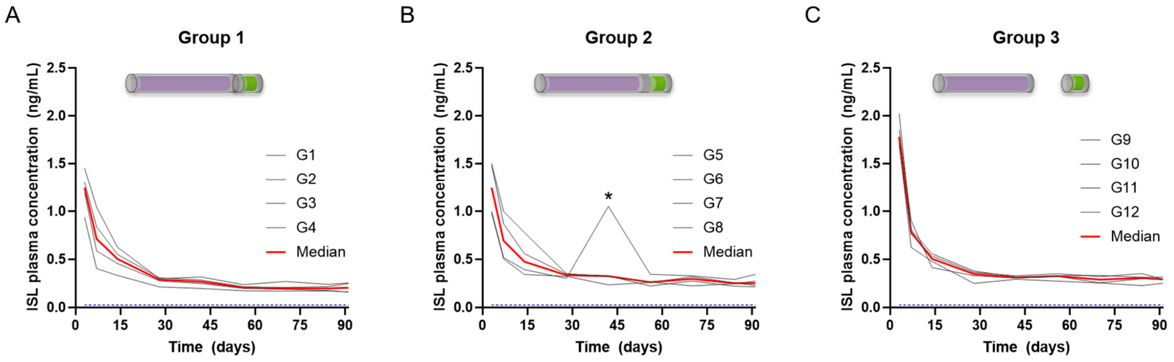

Figure S11. Individual ISL plasma PK profiles in NZW rabbits implanted with Groups 1, 2, and 3 MPT implants over the entire study duration (median shown as red line, n = 4 implants/group). ISL lower limit of quantification (LLOQ) shown as horizontal dotted line, 0.025 ng/mL. Asterisk (*) in panel B indicates an outlier for rabbit G7 on Day 42 (1.05 ng/mL ISL), exceeding the 1.5 × IQR upper threshold (0.415 ng/mL); included for variability but excluded from statistical analyses.

Table S3. PK parameters (median ± SD) calculated from mean plasma concentrations of ENG segments from Day 28-91 in NZW rabbits.

Group	Estimated ENG release rate (µg/day)	Estimated ENG dose level (µg/kg)	ENG C_{max} (ng/mL)	ENG $AUC_{(0-t)}$ (ng*d/mL)
1	39.1	14.3	0.76 ± 0.17	32.20 ± 4.2
2	40.9	13.5	0.89 ± 0.29	32.9 ± 20.2
3	33.2	11.4	0.66 ± 0.28	34.1 ± 14.7

Table S4. PK parameters (median ± SD) calculated from mean plasma concentrations of ISL segments from Day 28-91-day study in NZW rabbits.

Group	Estimated ISL release rate (µg/day)	Estimated ISL dose level (µg/kg)	ISL C_{max} (ng/mL)	ISL $AUC_{(0-t)}$ (ng*d/mL)
1	27.3	10.0	1.25 ± 0.22	29.7 ± 6.3
2	16.0	5.3	1.27 ± 0.0.28	37.0 ± 5.8
3	44.2	15.2	1.78 ± 0.15	37.9 ± 3.6

Table S5. Macroscopic evaluation of abnormalities (fibrosis, erythema or inflammation) in tissues surrounding the implant sites in the NZW rabbits at Day 91 as rated as the following: Severity: N = None (0); 1 = Minimal; 2 = Mild; 3 = Moderate; 4 = Marked; 5 = Severe

Group	Rabbit ID	Device ID	Encapsulation/Fibrosis	Inflammation or Erythema
1	G1	23	None to minimal fibrosis	None
	G2	26	Minimal fibrosis	None
	G3	29	Minimal fibrosis	None
	G4	32	None to minimal fibrosis	None
2	G5	45	None to minimal fibrosis	None
	G6	47	Minimal fibrosis	None
	G7	48	Minimal fibrosis	None
	G8	53	Minimal fibrosis	None
3	G9	13 (ENG)<br>3 (ISL)	None<br>Minimal fibrosis	None
	G10	14 (ENG)<br>9 (ISL)	Minimal fibrosis<br>Minimal fibrosis	None
	G11	16 (ENG)<br>8 (ISL)	Minimal fibrosis<br>None to minimal fibrosis	None
	G12	19 (ENG)<br>10 (ISL)	None<br>Minimal fibrosis	None
